# ‘A Most Protean Disease’: Aligning Medical Knowledge of Modern Influenza, 1890–1914

**DOI:** 10.1017/mdh.2012.29

**Published:** 2012-10

**Authors:** Michael Bresalier

**Affiliations:** Centre for the History of Science, Technology and Medicine, Simon Building, Brunswick Street, University of Manchester M13 9PL, UK

## Abstract

This article reconstructs the process of defining influenza as an infectious disease in the contexts of British medicine between 1890 and 1914. It shows how professional agreement on its nature and identity involved aligning different forms of knowledge produced in the field (public health), in the clinic (metropolitan hospitals) and in the laboratory (bacteriology). Two factors were crucial to this process: increasing trust in bacteriology and the organisation of large-scale collective investigations into influenza by Britain’s central public authority, the Medical Department of the Local Government Board. These investigations integrated epidemiological, clinical and bacteriological evidence into a new definition of influenza as a specific infection, in which a germ – *Bacillus influenzae* – was determined as playing a necessary but not sufficient role in its aetiology, transmission and pathogenesis. In defining ‘modern influenza’, bacteriological concepts and techniques were adapted to *and* selectively incorporated into existing clinical, pathological and epidemiological approaches. Mutual alignment thus was crucial to its construction and, more generally, to shaping developing relationships between laboratory, clinical and public health medicine in turn-of-the-century Britain. While these relationships were marked by tension and conflict, they were also characterised by important patterns of convergence, in which the problems, interests and practices of public health professionals, clinicians and laboratory pathologists were made increasingly commensurable. Rather than retrospectively judge the late nineteenth-century definition of influenza as being based on the wrong microbe, this article argues for the need to examine how it was established through a particular alignment of medical knowledge, which then underpinned medical approaches to the disease up to and beyond the devastating 1918–19 pandemic.

‘[S]uch phrases as the return of influenza, the reimportation of influenza, etc., are mere figures of speech; we have never lost it again since 1889’.^1^ So wrote the London epidemiologist, Major Greenwood. Like many medical observers, he was trying to contextualise the devastating 1918–19 pandemic. For Greenwood, the pandemic represented the culmination of a ‘new cycle’ in influenza’s history, which had begun when three epidemics swept the globe between 1889 and 1894. His view was that through its recorded history, influenza had appeared episodically, visiting Britain and Europe once or twice a generation, until the 1890s, when it became ‘endemic’ in industrial nations.^2^ The epidemics inaugurated what Greenwood called the ‘modern period’ of influenza, as it became ‘a factor of great importance in the causation of mortality’, and an inescapable part of modern life.^3^

Greenwood assumed, as did most of his contemporaries, that the signs, symptoms and pathology of influenza had been essentially constant through history and that what had changed since 1890 was the epidemiology of disease. In this article I show how, after 1889, medical practitioners were not simply mapping new behaviours of an old disease, but those of a new influenza. This new influenza was constructed principally with the ideas and tools of bacteriology and their integration into epidemiological and clinical knowledge and practices.^4^ Otto Leichtenstern, the Munich clinician and pathologist, explained this transformation in his influential 1898 manuscript on influenza. The medical profession across Europe, he noted, had confronted the pandemic as a ‘new disease’ and, by applying ‘the progress and the acquisitions of modern medicine, advanced…knowledge of influenza in every direction’.^5^ No longer a product of the air, atmosphere or cosmos, a new definition adhered to ‘the doctrine of the contagious nature of influenza, of its transmission from person to person, and its dissemination through human intercourse’.^6^

This article reconstructs the process of defining ‘modern’ influenza in the contexts of British medicine between 1890 and 1914. It shows how professional agreement on its nature and identity involved aligning different forms of knowledge produced in the field (public health), in the clinic (metropolitan hospitals) and in the laboratory (bacteriology). While there existed different influenzas in the wake of the 1889–90 pandemic, the problems, interests and practices of public health professionals, clinicians and laboratory pathologists were made increasingly commensurable, such that by the early 1900s influenza was generally characterised as a specific infectious disease.

Examining this process provides particular insight into the developing relationships between laboratory, clinical and public health medicine in Britain. Revisionist histories of the adoption and use of laboratory knowledge and practices have demonstrated the evolutionary rather than revolutionary nature of this process.^7^ However, as Steve Sturdy has recently argued, a strong tendency has been to examine this dynamic through the prism of the ‘essential tension’ between medical practice and medical science.^8^ Conflict has become the *status quo ante* in studies of the relations between the laboratory, the clinic and the field, with each domain characterised as having distinctive, if not incommensurable, institutional commitments, interests, values, skills, norms of practice and evidence.^9^ Sturdy argues that a consequence of this focus is that other kinds of negotiation, particularly those involving collaboration between laboratory and clinical practitioners, have been cast as exceptional rather than intrinsic to the making of modern medicine.

While the conflict model has been dominant, recent historical work has pointed to an important pattern of convergence, in which clinical and epidemiological knowledge shaped and was shaped by laboratory knowledge.^10^ This pattern has been well demonstrated for the integration of bacteriology into different realms of medical practice, and its role in changing definitions of disease at the end of the nineteenth century.^11^ Michael Worboys and Christoph Gradmann have shown, respectively, for instance, that to understand the redefinition of tuberculosis as a specific infectious disease in Britain and Germany in the 1880s, it is necessary to examine how bacteriological concepts and techniques were adapted into existing clinical, pathological and epidemiological approaches.^12^ Their studies suggest that the transformation of tuberculosis’s identity was the outcome of a process of mutual alignment and accommodation, in which bacteriological, clinical, pathological and epidemiological approaches to the disease were modified and made commensurable. Similar patterns have been identified in histories examining the relationship between the ‘bench’ and the ‘bedside’ in constructions of cardiac disease,^13^ cancer,^14^ aphasia^15^ and allergy.^16^

Studying patterns of convergence and alignment between the different professions, specialisms, disciplines, institutions and practices around which modern medicine has been built can yield a more rounded and empirically rich picture of the dynamics of producing medical knowledge, striking a balance between conflict and consensus. With this aim in view, this article approaches the process of defining influenza in late nineteenth-century British medicine as an example of how aligning different forms of knowledge was integral to creating the identity of a modern infectious disease. The first sections trace the respective ways in which public health, clinical and bacteriological studies defined influenza in the wake of the 1889–90 pandemic, stressing how each provided the other with conceptual and practical resources, and with specific problems to solve. The article then examines how evidence from these different lines of investigation was drawn together, and incorporated and used in public health, clinical medicine and pathology. Increasing trust in bacteriological ideas and methods in British medicine, especially after 1900, was a crucial factor in this realignment. Another significant factor was the organisation of large-scale collective investigations by Britain’s central public authority, the Medical Department of the Local Government Board (LGB). These investigations integrated epidemiological, clinical and bacteriological evidence into a new definition of influenza as a specific infection, in which a germ – *Bacillus influenzae* – was determined as playing a necessary but not sufficient role in its aetiology, transmission and pathogenesis. In defining ‘modern influenza’, bacteriological concepts and techniques were simultaneously adapted to *and* incorporated into existing clinical, pathological and epidemiological frameworks. The LGB was uniquely positioned to draw together such evidence and its relative success in doing so demonstrates the importance of paying attention to mutual alignment in the construction of a disease identity and, more generally, in the shaping of relationships between laboratory, clinical and public health medicine in turn-of-the-century Britain. While these relationships were certainly marked by tension and conflict, my analysis shows that they were also characterised by an important pattern of convergence in efforts to make sense of a highly protean disease.

Historians of influenza know that its late nineteenth-century definition as a specific infection was based on what was later shown to be the wrong microbe.^17^ Rather than a bacillus, its primary causative agent is now identified as a virus. But, as I argue below, retrospective diagnoses or judgements ignore the significance of the bacillus and bacteriology in redefining influenza and, crucially, how both figured in the realignment of clinical and epidemiological knowledge of the disease. This history is especially important if we want to understand medical approaches to the 1918–19 pandemic in Britain and elsewhere, precisely because they were based on the new ways of knowing influenza that were forged in the 1890s and 1900s. More generally, understanding the process of defining modern influenza highlights the particular importance of paying attention to processes of alignment and convergence in the production of medical knowledge at the turn of the nineteenth century.

## Public Health: Making Influenza Communicable

**Figure 1:**
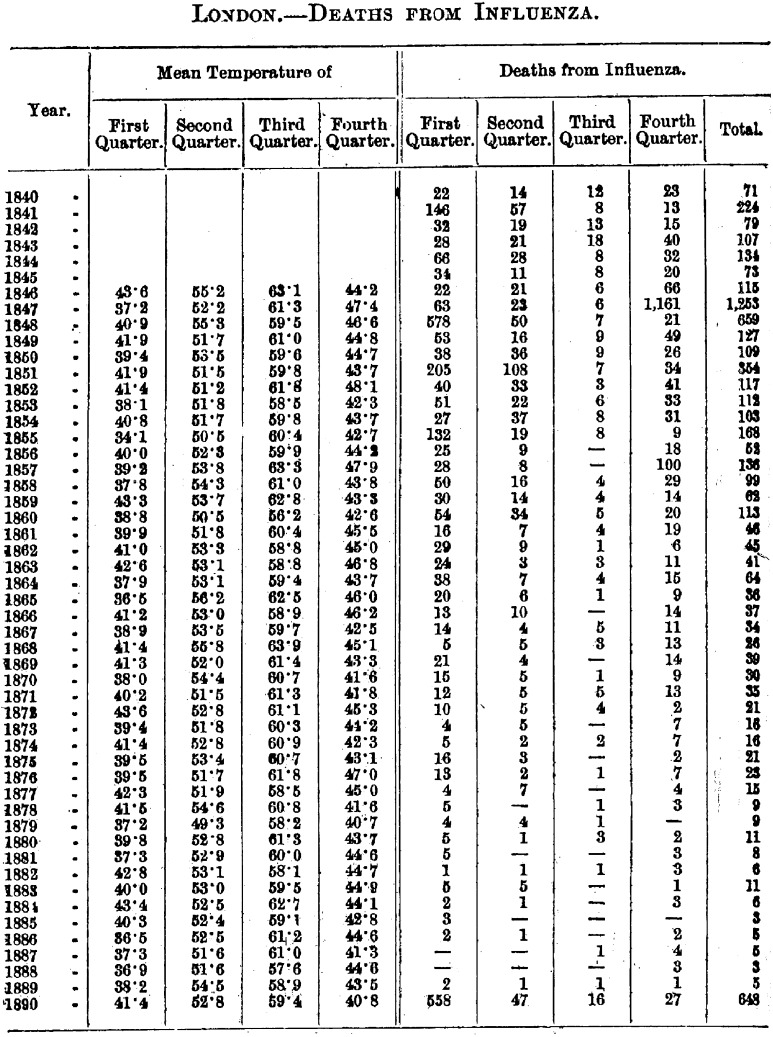
Deaths from influenza (London), 1840–90. Source: H.F. Parsons, *Report on the Influenza Epidemic of 1889–1890* (London: HMSO, 1891).

Through much of the nineteenth century the disease termed influenza played only a minor role in the affairs of government, medicine, public health and everyday experience. It existed on the fringes of Victorian life. London mortality statistics told part of this story. From when ‘influenza’ was first used as a term in English medicine in the 1740s to the 1850s, epidemics had visited London seven times.^18^ Large epidemics struck in 1782, 1801, 1830–1, 1833 and 1847–8.^19^ But after 1848, influenza slipped off the epidemiological map. For the next four decades, with the exception of small outbreaks in 1855 and 1858, its prevalence steadily declined. Between 1870 and 1888 it had almost disappeared from England and the rest of Europe (Figure 1).^20^

**Figure 2:**
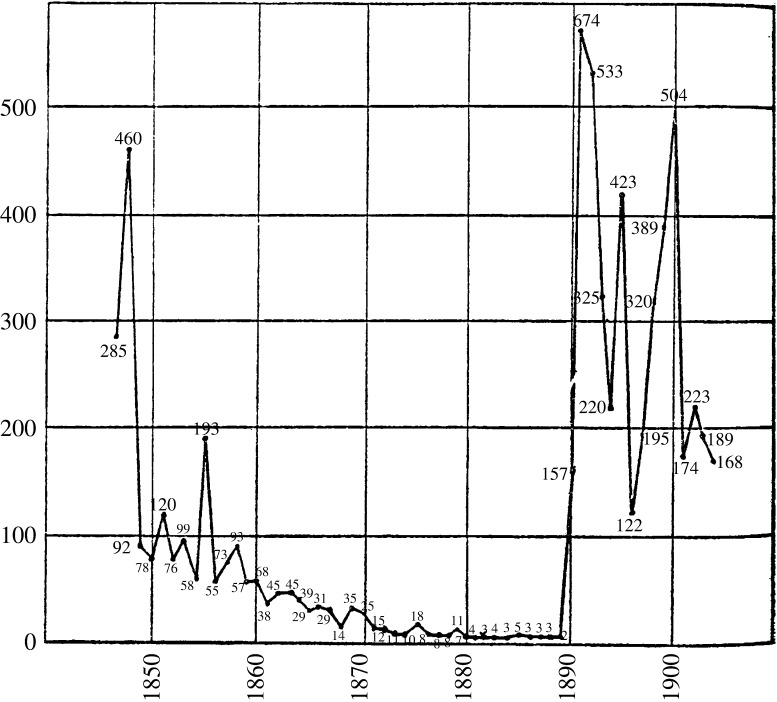
Annual influenza death rate per million in England and Wales, 1847–1905. Source: A. Newsholme, ‘Influenza for the Public Health Standpoint’, *The Practitioner*, LXXVII (1907), 118.

This period of decline came to an abrupt end in autumn 1889, when a massive epidemic swept across Europe and the rest of the world. Two ‘recrudescences’, one less widespread but more deadly in 1890–1 and another in 1892, followed this epidemic. Influenza killed no fewer than 57 980 in Britain in 1890 and 1891. Unlike in the past, however, it did not abate. In no year between 1890 and 1915 did fewer than 496 Londoners die from it; in ten of these years more than a thousand deaths were allotted to influenza. Major epidemics struck London in 1895, 1899–1900 and 1908–9.^21^ In the decade thereafter, the number of deaths averaged 11 050 per year, and never went below 3753 (Figure 2).^22^ The rest of Europe as a whole experienced a similar escalation.

Raw numbers underscored influenza’s changing epidemiological presence in the social experience of health and illness.^23^ But initially, while the epidemic was generally called ‘influenza’, neither public health professionals relying on epidemiological knowledge nor medical practitioners relying on clinical knowledge could agree on its actual nature. Agreement on its medical identity was the product of state-organised investigations that mapped and redefined its epidemiological, clinical and aetiological characteristics. During the 1889–90 epidemic, public health bodies across Europe organised large-scale studies of influenza, with the general aim of resolving questions about its origins, causes, modes of spread and what constituted a case of the disease. In Britain, the LGB’s Medical Department produced two widely influential reports.^24^ Drawing on methods of case-based epidemiology and the epistemological resources of bacteriology, these reports were widely heralded for producing a new way of understanding influenza. The first report, published in 1891, demonstrated the Department’s leading role in the use of modern epidemiological methods. The second, which we shall look at later, demonstrated their increasing interaction with bacteriological knowledge, and the Department’s key role in aligning epidemiological, clinical and laboratory evidence.

The Department’s reputation for epidemiological investigations was well established among European public health bodies.^25^ Before its investigations into influenza, bacteriology played a growing role in the production of epidemiological knowledge and, by the 1880s, the Department had taken up bacteriological practices as a standard part of its ‘Auxiliary Scientific Investigations’ into such diseases as typhus, diphtheria, smallpox, scarlet fever and tuberculosis.^26^ As part of this new orientation, the Department’s focus on ‘inclusive’ public health measures, guided by a sanitary vision and directed at the environment, increasingly gave way to ‘exclusive’ measures, specifically directed at disease agents, people and their interactions.^27^ Its investigations into influenza in the early 1890s were important to the process of integrating these changes into its approaches.

**Figure 3:**
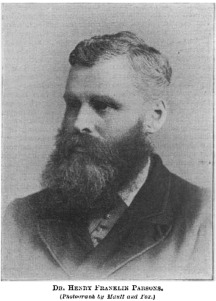
Henry Franklin Parsons (1846–1913). Source: *BMJ*, 8 November (1913), 1263.

At the height of the epidemic in December 1889, the LGB’s Medical Officer, George Buchanan, asked his assistant, Henry Franklin Parsons, to organise a ‘collective’ epidemiological investigation, with the aim of determining its origins and modes of spread – issues that sharply divided medical observers (Figure 3). Harry Marks has shown that collective investigations were first proposed by elite physicians in Britain as a way to transcend the study of diseases in hospitals and to involve general practitioners in reconstructing their ‘life-history’ through populations.^28^ A key aim was to standardise the production and use of medical knowledge, particularly diagnostic categories and practices. While, as Marks suggests, the collective investigation had limited popularity as a tool for clinical research, the basic principle of mobilising practitioners to study and to help generate a standard picture of a disease was one that the Medical Department put into practice in its own studies. Its style of collective inquiry, which it had already honed in investigations of cholera and smallpox, involved the entire public health system, gathering information from medical officers, GPs and other sources from across the nation and much of the Empire.^29^

**Figure 4:**
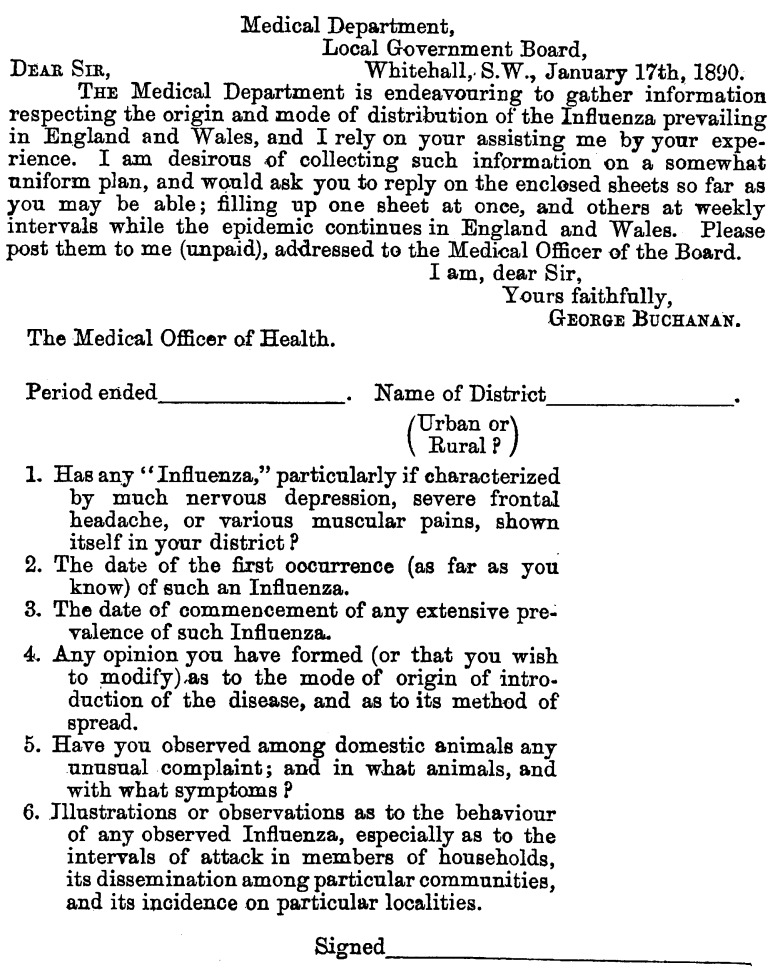
Questionnaire on the Origin and spread of influenza, 1890. Influenza is in scare quotes. Source: Henry Parsons, *Report on the Influenza Epidemic of 1889–1890* (July 1891), 120.

In early January 1890, Parsons issued a questionnaire to collect information on influenza on a ‘uniform plan’ (Figure 4). Case identification was key to determining the first occurrence of influenza and when it became epidemic. But it depended on an agreed clinical definition. The problem for Parsons was that most practitioners were unfamiliar with what constituted a case of influenza. While he provided a short list of standard symptoms based on older classifications, as we shall see, these did not match the ‘influenza’ practitioners actually encountered in 1889–90. Despite this obstacle, Parson proceeded to gather opinions on the ‘commencement’,  ‘the mode of origin of introduction of the disease’, ‘its method of spread’ and ‘dissemination’ through households, communities and localities.^30^ He sent the questionnaire to 1777 sanitary districts, to all government departments and heads of public and quasi-public bodies, medical and public health journals, and the daily press.^31^ Some 1150 reports were returned. Parsons’ first impression on reviewing them was ‘bewilderment. There is scarcely a single proposition made which was not contradicted by different observers.’^32^

The avalanche of reports revealed deep fissures on crucial questions about the influenza’s identity, the factors that caused the epidemic, how it spread and the speed it travelled. Part of the problem stemmed from the epidemiological complexity of the disease itself. Influenza’s ‘mode of diffusion stands almost alone among epidemic diseases’, the *Lancet* noted:

In the first place, it spreads with remarkable rapidity once it is established in a centre. Secondly, it tends more or less rapidly to become pandemic …its liability to diffusion over whole continents, and indeed from one hemisphere to the other, is one of the best-known facts concerning it. The disease therefore has no geographical limitation …and its virus travels over seas and land in manner so baffling and contradictory to the ordinary conceptions of the transmission of infection as to render any simple explanation of its nature almost impossible.^33^

But disputes over influenza’s epidemiology, and efforts to decide its clinical characteristics, were also rooted in competing medical epistemologies.

A priority for public health professionals was to explain how influenza had spread so rapidly and appeared so suddenly. At first, disagreement reigned. Some observers invoked theories based on the original meaning of ‘influenza’, which presupposed an external *influence* – occult, telluric, astral or meteorological – that conspired to excite an epidemic.^34^ The notion of an ‘epidemic constitution’, which was traced back to the seventeenth-century writings of the ‘English Hippocrates’, Thomas Sydenham, was widely used to argue that the epidemic was the product of one or a number of changes in temperature, moisture, air pressure, ozone levels and the nature and density of fogs.^35^ A long history of associating influenza with the weather had wide appeal because it resonated with popular perceptions of its apparent affinity for colder and damper months of the year. Reports in the general press attributed the epidemic to miasmas activated by elemental forces, such as floods, droughts, earthquakes, volcanoes or electrical magnetic waves.^36^

Historians have focused on rivalries in epidemiological thinking between supporters of miasmatic and contagion theories, but, as Pelling has shown, in practice the dominant view in England was that of contingent contagionism and there were different models for different diseases.^37^ By 1890, bacteriological ideas were reshaping, albeit unevenly, epidemiological models, with more emphasis on the modes by which diseases were transmitted and the role of living pathogens in epidemic diseases.^38^ At the beginning of the epidemic in January 1890, a *British Medical Journal* (BMJ) editorial argued that, ‘like other epidemic diseases, influenza is spread by a contagium, and must be due to a living organism, a microbe’, but there remained dispute over whether the stunning diffusion of influenza was the product of ‘microbes carried in the air’, microbes spread from one person to person or a combination of the two.^39^ For many observers, influenza defied explanation as a strictly contagious disease. They concurred with mid nineteenth-century observations that conceptualising influenza as a contagion could not account for the speed at which epidemics spread, how they struck countries ‘as if at one blow’ and extended over ‘the whole of the inhabitable globe’.^40^ Early reports reinforced this view of the 1890 epidemic. ‘The most remarkable phenomenon of [the epidemic’s] progress’, noted the *Lancet*, ‘was the rapidity, almost suddenness, with which large numbers of persons, or entire communities, were attacked, exceeding anything observed in the case of other infectious diseases, and not easily explicable on the ordinary theory of contagion, but apparently suggesting an aerial conveyance of some kind’.^41^

**Figure 5:**
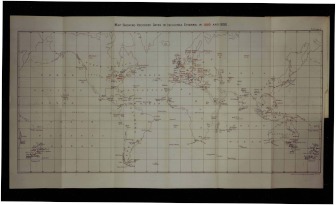
Global Map of 1889–90 Influenza Epidemic. Bokhara, in the Russian steppe, was identified as the geographical origin of the epidemic. Source: Henry Parsons, *Report on the Influenza Epidemic of 1889–1890* (July 1891).

Henry Parsons’ *Report on the Influenza Epidemic of 1889–1890*, published in July 1891, reflected on the different aetiological theories aired during the epidemic and concluded that the evidence supported a new contagionist position. He wrote that influenza was ‘an eminently infectious complaint, communicable in the ordinary personal relations of individuals, one with another’, in cities, institutions and homes.^42^ The *Report* defined influenza as a disease that spread on the ‘lines of human intercourse’. It posited a ‘germ’ as the contagion, which was communicated directly from person to person, and it inferred that, because its incubation period was short it spread more rapidly and extensively than any epidemic disease.^43^ Tracked in this way, Parsons was able to map influenza’s spread across the world (Figure 5).

The Report was much anticipated and well received. The *BMJ* noted that although ‘the theory that influenza is mainly if not entirely spread by contagion is no new one …[it] had needed to be born again.’^44^ In a significant turn, the *Lancet* endorsed the new concept: ‘there does appear to be an abundance of evidence to show that [the epidemic] travelled mainly along the lines of human intercourse, attacking large towns and population centres first …and that the disease travelled only just as fast as any humanly conveyed infection …might have expected to travel’.^45^ A survey of London Medical Officers found that only one of nineteen held that influenza was a miasma; another remained undecided; the rest supported its identity as an infectious disease.^46^ In late January 1892 the Society of Medical Officers of Health passed a resolution defining influenza as a ‘dangerous infectious disease’^47^ and considered including it under the 1889 Infectious Diseases Notification Act.^48^ Reports by German, French and other European health authorities confirmed the LGB’s conclusions.

Influenza was drawn into a new discourse that associated risk of infection with specific conditions of the modern city. Catching and spreading influenza was attributed to increasing urban interconnection and propinquity. ‘The assembly of people in churches, chapels, the Houses of Parliament, places of business, or other institutions’ involved what the *Lancet* described as the ‘exceptional risk of contracting the disease, the risk depending in part on the closeness of aggregation’.^49^ Subsequent epidemics in 1890–1, 1892 and 1893–4 confirmed this general picture. They demonstrated how modernised cities fostered conditions that made influenza endemic. People living in close proximity or overcrowded conditions, and increasingly concentrated in ever-larger industries, offices and institutions, provided constantly fresh soil for influenza.^50^ The centrifugal growth of suburbs, facilitated and serviced by railways, created a continuous flow of people travelling in and out of cities, and keeping influenza in constant circulation. What Greenwood later described as the ‘steady increase of movement and intermingling of populations associated with the improvement of communications’, made influenza intractable.^51^

In this framework, the ecological conditions of the modern city and communications fostered influenza and its dangers. Yet unlike with cholera or tuberculosis, which sharply revealed class divisions, this risk was presented as being shared by all. Influenza was, in the words of the *Times*, an ‘essentially democratic’ disease that embraced ‘all the people, not just the lower section of them’.^52^ The concept of influenza as ‘democratic’ became a trademark of its modern identity. It was closely tied to its definition as an infectious disease, which placed it under the aegis of preventive medicine and its focus on germs, people and the places in which they interacted. Crucially, however, it was the fact that influenza eluded prevention that democratised its danger. ‘Theoretically’, noted Louis Parkes in his manual on *Hygiene and Public Health*, ‘notification of cases, isolation of the sick, and disinfection of premises, should be, as for other infectious diseases, the proper means of checking or stamping out an epidemic’.^53^ But there was considerable doubt about the effectiveness of these measures against influenza. In his introduction to Parsons’ report, the LGB’s Medical Officer, George Buchanan, admitted that, ‘from what we have thus far seen of the specialities of influenza, we cannot feel particularly confident, under the existing conditions of society, to successfully defend ourselves against a further outbreak’.^54^ Isolation was unimaginable during epidemics: ‘When two-thirds of the households, and perhaps not far short of a fourth of the adult population, are suffering from the disease’, asked the *BMJ*, ‘is isolation of the sick anything more than a dream?’^55^ Influenza’s incubation rate was so short that it became rapidly infectious in its early stages and, coupled with its non-specific symptoms, this meant practitioners had great difficulty recognising first cases. While disinfection might have limited influenza’s spread, no one knew what to disinfect.

Public health authorities needed answers to two crucial questions: What constituted a case of influenza and what was its disease agent?^56^ For these, they turned to clinicians and bacteriologists.

## Clinical Medicine: Proteus in the Clinic

Parsons’ first report relied on descriptions of influenza supplied by GPs and medical officers, but it revealed significant gaps in existing clinical knowledge. Few practitioners had experience identifying or managing influenza. It did not fall under the 1889 Infectious Diseases Notification Act, so doctors were not obligated to report it.^57^ Since it had not registered as a serious medical problem for decades, most practitioners had no reason to learn how to recognise its signs and symptoms, or to distinguish these from other diseases. This not only challenged epidemiological understandings, but it also had implications for medical practice.

Without first-hand experience of influenza, practitioners were forced to turn to existing textbooks for guidance. Yet the descriptions they found had not been revised since the 1850s. Significant gaps emerged between physicians’ own observations and the *status quo* of an earlier generation. One striking discrepancy was the relative absence of ‘catarrh’ in 1890.^58^ Following a practice that could traced back to William Cullen’s classification of influenza as a ’*catarrhus a contagio*’ in the 1780s, clinical accounts of the 1830s and 1840s made catarrh – general inflammation of the respiratory tract – a defining symptom, and textbooks categorised influenza as an epidemic ‘catarrhal fever.’^59^ In contrast, physicians in the 1890s highlighted the predominance of ‘respiratory’ and ‘nervous’ symptoms. Such gaps raised questions about the state of medical knowledge: If older textbooks were out-dated, what constituted a reliable basis for diagnosis?

Physicians attributed the change in influenza’s predominant symptoms to its clinical variations, a fact demonstrated in historical records and medical observations. But gaps in clinical knowledge also stemmed from significant changes in the social organisation and epistemology of medicine since ‘epidemic influenza’ last appeared in 1848. To learn how to identify influenza, the profession had to re-shape its clinical identity to fit contemporary medical knowledge and practice. The most significant change was from defining influenza as a species of ‘catarrh’ to its identification as an acute disease in which catarrh and fever were symptoms of a respiratory infection.

The practical transformation of influenza’s clinical identity was rooted in London’s voluntary and teaching hospitals, where elite clinicians codified clinical knowledge and practice.^60^ Although GPs and medical constituencies developed their own ‘influenzas’, clinicians had the power to produce a standard clinical picture. The availability of hospital patients made it possible to observe and classify the varieties of influenza, to follow its pathogenesis and to analyse its morbid anatomy and clinical pathology. Increasing technological sophistication of hospitals provided clinicians with a range of tools with which to trace influenza’s extensive constitutional symptoms and complications. Most crucial was thermometry, which enabled clinicians to standardise fevers associated with particular diseases.^61^ Hospitals drew together different kinds of knowledge in one institution.^62^ The epidemics demonstrated the extent to which the production of medical knowledge was becoming collective.^63^

At the outset of the 1889–90 epidemic, London’s leading medical bodies called upon physicians to put the production of ‘exact clinical records’ at the forefront of the medical agenda. The *Lancet* led the push: ‘[W]e…suggest, in the interest of scientific medicine, that, as nearly half a century has elapsed since the last outbreak of the disease in England, no means should be left untried to ensure full records of the occurrences of cases 
’.^64^ Leaders of the profession saw the pandemic as an opportunity to order the clinical picture of influenza. Standardisation of diagnoses had become a platform for those seeking reform.^65^ It represented a way to unify and assert control over practices and meanings assigned to diseases, and especially for one that showed as much heterogeneity as influenza.

But rather than clarify influenza’s clinical picture, the call to collect new information initially complicated it. As the first epidemic reached its apex in early January 1890, clinical descriptions filled the pages of the medical and general press.^66^ Physicians identified a profuse number of symptoms in the patients they saw. Richard Sisley, a Harley Street physician, surmised that, ‘to sum up accurately all the symptoms of influenza in a single sentence is impossible’.^67^ James Goodhart, a physician at Guy’s Hospital, insisted that influenza stood above all diseases for ‘the width of its range of action over the human body…. There would appear to be no organ or tissue that has not become the subject of its attack’.^68^ The breadth and lack of specificity of symptoms corresponded to numerous clinical interpretations.

The lack of an agreed diagnosis worried the medical establishment. Elite physicians blamed GPs’ lack of training and discipline for these difficulties. At the height of the 1889–90 epidemic it was not unusual for a GP to see two or three hundred patients a day. The majority had only brief encounters; their aim was to provide rapid diagnoses and treatment. Critics claimed that in their haste GPs pandered to diagnostic ‘fashions’ and used ‘popular symptoms’ derived from the general press or patients. This proliferated clinical definitions, added confusion to a complex problem and threatened professional authority.^69^

The elaboration of influenza’s clinical identity at St Bartholomew’s Hospital in the early 1890s exemplifies how a general diagnostic framework was built.^70^ While influenza was never the property of one institution, Barts established itself as an important centre of clinical and pathological investigations. Samuel West organised these studies. Oxford educated, he was Assistant Physician to Barts and Physician to the City of London Hospital for Diseases of the Chest. His knowledge of chest diseases put him in a good position to clarify influenza’s respiratory complications. An exponent of the idea that influenza was primarily a respiratory ailment, he was among the first to characterise ‘influenzal pneumonia’, an important complication. Barts was a major destination for the sick during the 1889–90 epidemic. West estimated in the first six or seven weeks of 1890 ‘not far short of 8000 cases of influenza were seen and treated’ in the Hospital’s Casualty Department and wards.^71^ The majority came in the second and third weeks of January, when nearly 6000 patients visited – two-thirds suffering from influenza. The epidemic temporarily paralysed the hospital.

**Figure 6:**
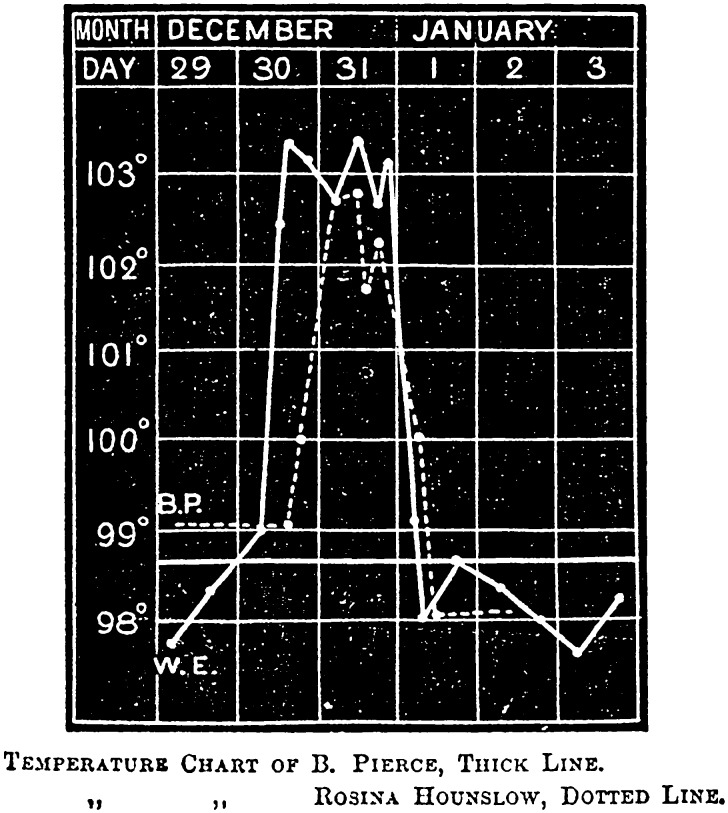
‘Uncomplicated influenza’. Fever chart of Bedford Pierce, 28, and Rosina Hounslow, the first recognised case at Barts, 30 December 1889. Source: S. West, ‘The Influenza Epidemic of 1890’, *St. Bartholomew’s Hospital Reports*, XXVI (1890), 212.

**Figure 7:**
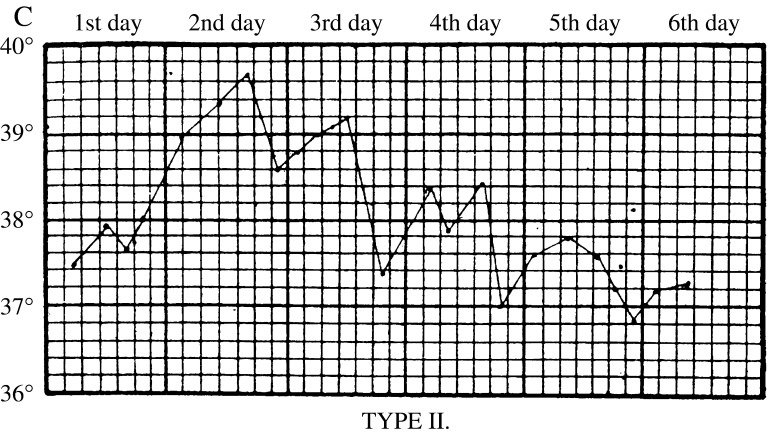
Relapsing fever. A standard reference was Otto Frentzel, ‘Zur Kenntnis des Fieberganges bei Influenza’, *Centralblatt fur klinische Medicin* (11 January 1890), who characterised three types of fever epidemic in the Municipal General Hospital at Friedrichshain. Type II, shown here, was a relapsing fever that could last for a week, with significant temperature fluctuations, in which the patient might appear to recover, only for the fever to return. Source: J.W.S. Moore, ‘Influenza’, in J.W. Ballantyne (ed.), *Encyclopaedia Medica* (Edinburgh: Green & Son, 1919), 517

**Figure 8:**
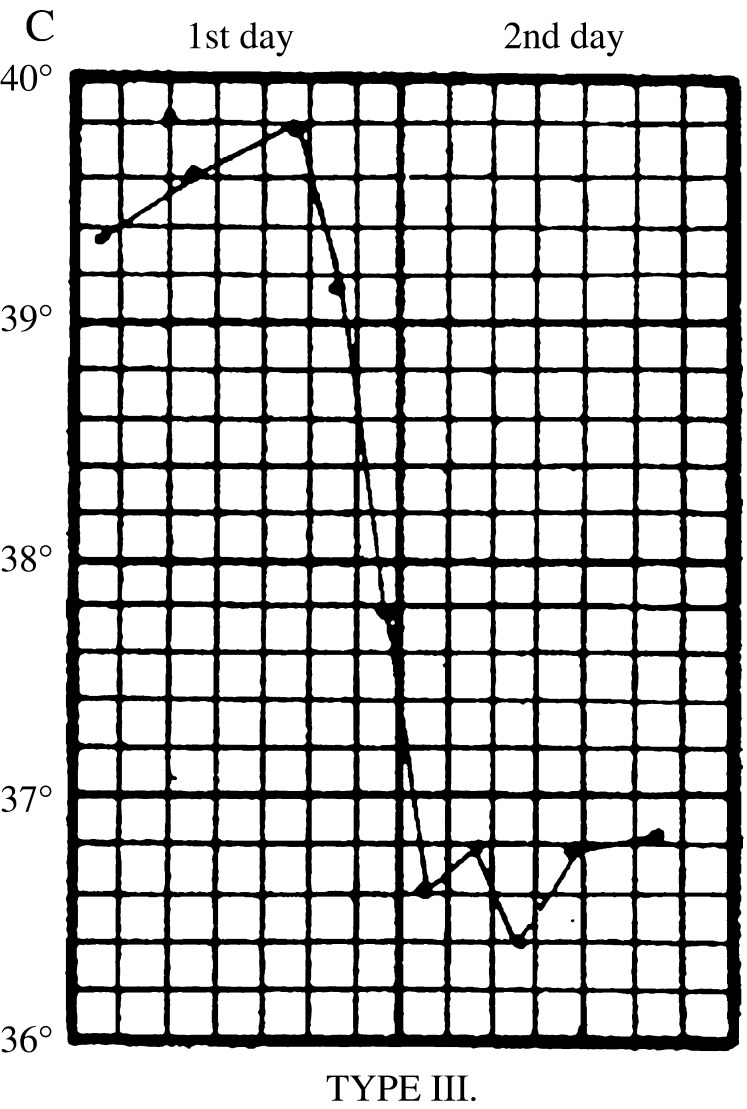
Apyretic fever. From Otto Frentzel, ‘Zur Kenntnis des Fieberganges bei Influenza’, *Centralblatt fur klinische Medicin* (11 January 1890). Type III ‘apyretic’ fever, marked by a sudden fall in temperature below normal, indicative of the onset of serious secondary complications, especially pneumonia. Source: J.W.S. Moore, ‘Influenza’, in J.W. Ballantyne (ed.), *Encyclopaedia Medica* (Edinburgh: Green & Son, 1919), 517

In the first stages of the epidemic, early symptoms were so indistinct that house physicians failed to identify them. John Ogle, a junior houseman, confessed that he was initially unable to ‘recognise the nature of the disease’; he and his colleagues thought it was ‘pneumonia, scarlet fever, or rheumatic fever’. Early attacks ‘remained an enigma until the epidemic arrived in full force’.^72^ Along with physical examinations and urine analysis, key information came from daily temperature readings, which clinicians used to chart what came to be known as ‘influenzal fever’. Corresponding to the acute stage of the disease, it involved a sudden rise in temperature at the onset of influenza, which peaked after forty-eight hours, and then fell back to normal within a few days (Figure 6). The fever gave clues to significant events and changes. A ‘relapsing’ fever, in which the patient’s temperature would rise, fall and rise again, was common (Figure 7). Most worrisome was a sudden drop in temperature to below normal after the acute stage, called ‘apyretic influenza’, for this often signalled the onset of more severe respiratory complications (Figure 8).^73^

Thermometry yielded new physical signs that became crucial to the prognosis of influenza. But clinical tools could not identify a single pathognomonic marker on which to anchor diagnosis. Clinicians resorted to grouping symptoms into patho-physiological forms. This practice had started in the early nineteenth century. During the 1848 epidemic, for instance, the London physician, Thomas Peacock, divided influenza into ‘simple catarrhal fever’, ‘catarrhal fever with pulmonary complications’ and ‘catarrhal fever with abdominal complications’.^74^ Theophilus Thomson, a leading physician at the Brompton Hospital for Consumption, did the same in his 1850 *Annals of Influenza*, the first historical chronicle of the disease.^75^ Clinicians in the 1890s modified these classifications.

Influenza was typically distinguished into ‘simple’ or uncomplicated and complicated cases. West characterised simple influenza as an acute disease, with an abrupt onset, in which ‘the temperature …rose at once’ and the patient was suddenly crippled by severe prostration, involving a range of constitutional symptoms, the most prominent of which were intolerable pain in the limbs, loins, eyes, back, spine and forehead. As the fever increased, ‘patients lay in a dull, heavy, drowsy state, though sleep was unrefreshing and disturbed by dreams, or even slight delirium’.^76^ Clinicians grouped symptoms according to how they affected the nervous system, respiratory or alimentary organs.^77^ West identified four varieties: a ‘*simple uncomplicated febrile*’ form, characterised by mild respiratory symptoms and influenzal fever, which rose quickly in the first forty-eight hours and subsided after three or four days; a ‘*gastro-intestinal form*’, marked by mild gastric symptoms in addition to fever and prostration; a ‘*catarrhal form*’, which was ‘singularly infrequent’; and a ‘nervous’ form, which involved excruciating neuralgic pains that produced debilitating paroxysms.^78^

As clinicians around London produced similar classificatory schemes, there was a recognised need to make them commensurable. In 1890, the *Medical Annual*, an indispensable tool for general practitioners that synthesised current medical knowledge into a standard reference, recommended that influenza be grouped into four clinically distinct forms: ‘simple catarrhal fever’, pulmonary, gastro-intestinal and nervous.^79^ Versions of this scheme quickly became part of clinical descriptions and of textbook medicine.^80^

Prevailing medical and social concerns shaped the taxonomy and meanings ascribed to key symptoms.^81^ New categorisations of ‘nervous’ and ‘respiratory’ diseases proved especially popular. Both were major factors in contemporary experience of health and disease. During the 1890s, practitioners were fixated on the astonishing range of neurological symptoms associated with influenza. They described patients affected with everything from mild neuralgia and neuritis to hypochondria, melancholia, mania and general paralysis. They were especially drawn to the extensive nervous and muscular depression influenza induced. ‘All at once all energy and vigour vanish; the patient feels for nothing and cares for nothing, is completely apathetic, listless, and gloomy, and may hardly have strength enough even to be irritable or sulky’.^82^ Nervous exhaustion – ‘neurasthenia’ – which had its own recent history became a defining feature of influenza, supplanting catarrhal symptoms. Specialists in nervous diseases argued that neurological symptoms indicated influenza’s propensity to attack the nervous system. Julius Althaus, Senior Physician to the London Hospital for Epilepsy and Paralysis, likened influenza to neuroses seen in syphilis.^83^ Benjamin Ward Richardson, a senior physician and author of the utopian sanitary critique, *Hygeia, or City of Health*, described influenza as an ‘epidemic neuroparesis’ that directly affected ‘organic nervous function’ and produced ‘intense depression’ in patients.^84^

Althaus, Richardson and others linked nervous influenza to the demands of modern life. People exhausted ‘by age, by work, by nervous excitability, by late hours, by anxiety, by strain mental or physical, by confinement in close rooms, by broken rest’ were the most susceptible, while the ‘nervously exhausted’ were the most stricken. Influenza worsened existing neuroses and produced a raft of ‘nerve invalids’.^85^ ‘Grave neurasthenia’ and ‘grave mental disorders’ remained for weeks or became permanent conditions.^86^ Bouts of insanity, suicides and murders were blamed on influenza’s nervous sequelae. Wilfred Harris, a colleague of Althaus’, remarked that, ‘there is scarcely a disease of nerve cell or fibre that has not been ascribed to this most searching of diseases since the last great pandemic of the early nineties’.^87^ Nervous influenza highlighted *fin de siècle* obsessions with moral and physical weakness, uncertainty, vulnerability, irrationality and sudden death.^88^

But nervous diseases were more a symbolic than an epidemiologically significant danger. Chronic and acute respiratory diseases such as tuberculosis, bronchitis and pneumonia were far more formidable.^89^ In the spectrum of complications, noted West, ‘the affections of the respiratory organs’ held ‘first place’.^90^ The most insidious was how easily influenza morphed into serious respiratory conditions. In a matter of days, a simple case could be supplanted by bronchitis, bronchopneumonia, lobar pneumonia, pleurisy and endocarditis. Influenza paved the way for dangerous respiratory problems, including tuberculosis. Patients with existing pulmonary tuberculosis were especially vulnerable. Respiratory complications often prolonged influenza in previously healthy individuals and often killed ‘those enfeebled by ill-health or age’.^91^

Clinicians most feared the signs of influenza moving into the lungs, as this signalled the beginnings of pneumonia. Among the deadliest of nineteenth-century respiratory diseases, pneumonia was influenza’s gravest complication. Different kinds of pneumonia were evident in influenza. Bronchopneumonia was the most prevalent. Clinicians also identified a specific ‘influenzal pneumonia’. West led the way in characterising this complication. Studying hundreds of patients at Barts between 1890 and 1894, he noted that although pneumonic complications could appear at the onset, influenzal pneumonia often followed convalescence. It could proceed from bronchitis or attack the lungs directly. Men were more susceptible than women, in part because they often returned to work after the initial fever had fallen and, without having fully recovered, made themselves vulnerable. While it presented typical pneumonic signs – raking cough, crackling rales, chest pain and asthenia – in other respects it was ‘atypical and eccentric’.^92^

For all their work, physicians still had trouble determining a case of influenza on symptoms alone. Although new classifications ensured continuity in clinical descriptions and became part of textbook medicine, they did little to relieve practical difficulties. By illuminating influenza’s protean identity, they also multiplied the number of possible symptoms and complications that could fall under the name. With no specific sign, physicians relied on influenza’s epidemiological characteristics to direct their clinical observations. In the second edition of his *Principles and Practice of Medicine*, the great Canadian clinician William Osler noted that, ‘when [influenza] occurs in epidemic form’ it could be easily identified by its ‘sudden onset, short duration, acute fever, great prostration, more or less severe catarrh, neuralgic pains, or gastrointestinal disturbance and the disease’s tendency towards severe respiratory complications’.^93^ But he warned against making hasty diagnoses outside of recognised epidemics, when influenza could be easily conflated with other respiratory ailments. Because of the threat of complications, Osler insisted that, ‘in every case the disease should be regarded as serious, and the patient should be confined in bed until the fever has completely disappeared’.^94^

## Bacteriology: The Influenza Germ

Part of the diagnostic challenge related to practitioners’ inability to identify influenza’s cause. The *BMJ* summed up the practical constraints: ‘A rational systematic mode of treatment is of course impossible, so long as the aetiology remains obscure, and hence we have at present to rely upon the main symptoms for indications’.^95^ An apparent solution emerged from the laboratory of the Berlin bacteriologist, Richard Pfeiffer, in January 1892, when he identified a new bacillus as the cause of influenza. Many hoped that this would facilitate new cause-based approaches. Certainly, the incorporation of the bacillus into the discourses and practices of clinical and public health medicine established the laboratory as a new site in the negotiation of influenza’s medical identity.

Historians treat Pfeiffer’s bacillus as the wrong aetiological agent around which an entire generation organised its knowledge. Retrospective assessments of Pfeiffer’s research point to flaws in his methods and interpretations, and the failure of the bacillus to meet Koch’s postulates. ‘No organism’, wrote the London pathologist, Robert Donaldson, in an authoritative review of the bacteriology of influenza in 1922, ‘has had such wide acceptance on grounds so insufficient as Pfeiffer’s so-called *Bacillus influenzae*’.^96^ The historian of influenza Alfred Crosby described Pfeiffer’s bacillus as ‘an authoritative road sign pointing in the wrong direction’.^97^

It is worth remembering, however, that the bacillus was represented, investigated, mobilised and disputed as the primary cause of influenza for nearly forty years, from the mid-1890s to the early 1930s. In medical, hygiene and bacteriology textbooks it was viewed as central to influenza’s definition as a specific infectious disease.^98^ Rather than dismiss the bacillus as an erroneous object, we need to understand how it gained legitimacy as influenza’s causative agent and how it was employed in laboratory, clinical and public health medicine.

**Figure 9:**
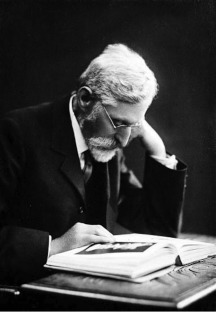
Edward Emanuel Klein, MD, FRS (1844–1925). Source: Wellcome Library.

The fate of Pfeiffer’s bacillus in Britain was intimately linked to the work of the LGB’s Medical Department and the medical press, particularly the *BMJ* and the *Lancet*. Edward Klein, the Vienna-trained histologist and reputed ‘father’ of British bacteriology, headed the Department’s investigations. Between 1892 and 1893 he confirmed Pfeiffer’s aetiological claims. Legitimising Pfeiffer’s bacillus, the Department also legitimised the role of the laboratory in defining influenza (Figure 9).

During the 1889–90 epidemic, eminent researchers in Berlin, Vienna and Paris, and other European capitals proposed a number of agents as the ‘influenza bacillus’. No fewer than eight different microbes made headlines.^99^ Pfeiffer’s bacillus was not among them. All were known germs, but none could be found in all cases. The *BMJ* and the *Lancet* cautioned against accepting any as the cause of influenza.

The reception of Pfeiffer’s bacillus contrasted sharply with these evaluations. Pfeiffer announced his discovery in Berlin on 4 January 1892, and it was widely heralded as a major breakthrough. The *Lancet* proclaimed that the discovery would ‘advance …knowledge of this mysterious plague’.^100^ The *BMJ* reproduced Pfeiffer’s preliminary report and a supporting paper from his colleague, Shibashuro Kitasato. A week later, it printed ‘*Some Remarks on the Influenza Bacillus*’, by Klein, who described how he had isolated and visualised a bacillus from the sputum of patients that ‘completely coincides with and confirms what is described by Pfeiffer and Kitasato’.^101^ The following week, the *BMJ* commented that the Berlin bacteriologists’ reports, along with Klein’s work, supported ‘the assertion that the problem as to the microorganism of influenza has at last been solved’.^102^

In response to Pfeiffer’s reports, in early January 1892 the Royal College of Physicians and the LGB discussed obtaining a Royal Commission to investigate the pathology of influenza, with a focus on Pfeiffer’s bacillus. In lieu of the commission, the Medical Department organised a second large-scale investigation in February 1892.^103^ In addition to new epidemiological work, it aimed at developing ‘more authentic modes of identifying influenza proper’ and gaining ‘better insight into the character, habits, and conditions of multiplication of the material of influenza’.^104^ Headed by Klein, it was the first state-based bacteriological study of influenza in Britain.

Since bacteriology was still on the periphery of metropolitan medicine, a tacit goal of Klein’s inquiry was to establish its medical relevance. More than just a means to isolate disease germs from sick bodies, bacteriological practices were constituted as necessary for the elucidation of the pathological properties of the influenza bacillus and in turn its clinical and epidemiological presentation.^105^ Explaining pathological processes through the proliferation and distribution of bacteria inside and outside the body was a prerequisite of bacteriological thinking.^106^ By identifying such properties, Klein linked Pfeiffer’s bacillus to the disease observed by clinicians and medical officers.

Klein’s laboratory was comprised of a series of pathology and histology rooms spread between the Brown Institution, the College of State Medicine and Barts, where he had two small workrooms in the Department of Pathology.^107^ These institutional constraints affected the scale of his inquiry. Whereas Pfeiffer allegedly had access to hundreds of cases, Klein acquired clinical material from fifty-six patients. Most came from the Kensington Infirmary in south-west London, while a few samples were collected at the outpatient’s clinic at Barts and Dr Barnardo’s Home, London’s infamous boys’ orphanage.^108^ The laboratory work was all done at Barts, where Klein recruited former pathology students, including F.W. Andrewes, to aid him. As it turned out, Klein himself suffered a bad case of influenzal pneumonia and passed the work to Andrewes.

The Department’s inquiry was modelled on the Berlin workers’ preliminary publications.^109^ Pfeiffer reported first identifying the bacillus in November 1891 on cover-glass preparations of purulent sputum spread. When dried and stained the bacilli appeared under light microscope as ‘very tiny rodlets’, often strung together in chains of three or four.^110^ Kitasato isolated the bacilli in pure cultures on sloping surfaces of glycerine-agar, where they appeared as minute, translucent colonies that looked like little water droplets.^111^ Cultured bacilli were readily distinguished from other microorganisms. Since Pfeiffer was unable to isolate them in other respiratory conditions, he surmised that they were specific to influenza. But Pfeiffer and Kitasato also ran into difficulties. Although establishing cultures was relatively straightforward, they had trouble keeping them alive. Even more vexing, they were unable to reproduce the disease in an experimental animal. Pfeiffer and Kitasato rather ingeniously accounted for these problems. Pfeiffer argued that the lack of a research animal indicated that, like cholera, influenza was specific to humans. Kitasato suggested that the distinctive culture characteristics of the bacillus could be used as markers for its identification. Initially overlooked, these problems later became important to debates over the aetiological status of Pfeiffer’s bacillus.

As the third epidemic in London began to wane in early February 1892, Klein and Andrewes adopted the Berlin workers’ methods. They tested Pfeiffer’s claims with bronchial samples from twenty patients, and one lung sample from a victim of influenzal pneumonia.^112^ Their experiments supported Pfeiffer’s argument that the respiratory tract was the primary locus of infection.^113^ Using cover-glass preparations of stained sputa they identified his bacillus in all of their samples. In many cases, it was visible in clusters and twisted chains among ‘crowds of other bacteria’ including streptococci and diplococci.^114^ Over half of the preparations contained an abundance of bacilli, often in almost pure culture. From these specimens, Andrewes produced broth and glycerine-agar cultures, in which he isolated the bacillus in colonies identical to those described by Pfeiffer and Kitasato. Like the Berlin workers, he was unable to reproduce the disease in experimental animals; however, he was able to sub-culture the bacillus in broth tubes for numerous generations.^115^ Andrewes’ experimental work confirmed the identity of Pfeiffer’s bacillus and its presence in the sputa of influenza patients. Yet, this only fulfilled one aim of the study; the key task was to determine whether the bacillus provided a better means of identifying influenza. This problem figured centrally in Klein’s official report.

Published in September 1893, it correlated three kinds of evidence: case reports of patients from whom clinical material was taken; laboratory studies that rendered the bacillus visible in this material and determined its properties; and photomicrographs of the bacillus in preparations from different body parts at different stages of the disease. By connecting the clinical picture to the properties of the bacilli, Klein produced what he described as ‘the aetiology of influenza’.^116^

**Figure 10:**
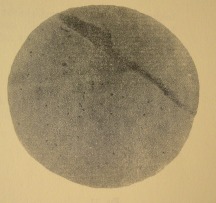
Walter Hall’s Pfeiffer bacillus. Cover-glass specimen showing ‘an almost pure culture of the specific bacillus’. Source: E. Klein, ‘Report on influenza in its clinical and pathological aspects’, in Parsons, *Further Report and Papers on Epidemic Influenza, 1889–92* (London: HMSO, 1893), 120

Correlating bacterial and clinical pictures involved using exemplary cases, such as that of Walter Hall.^117^ An eighteen-year-old butcher’s assistant, Hall was admitted to Kensington Infirmary on 28 January 1892, where the attending physician took his clinical history. Nine days earlier, he had come down with sudden and intense head pains, followed by shivers, which forced him to miss five days of work. He then had a severe relapse after going back, and ended up at the Infirmary. The relapse reached its height on 3 February, when his fever spiked and his doctor reported signs of respiratory complications. A day earlier, Klein made cover-glass preparations and cultures of Hall’s bronchial sputum. He observed ‘an almost pure culture of the specific bacillus’ when he examined the microscope preparations, while on agar-culture he isolated and produced the characteristic chains of the bacilli. Photomicrographs demonstrated the results of both techniques and helped to identify and trace the pathogenesis of the disease (Figure 10). Klein associated the presence of influenza symptoms with the presence of an abundance of bacilli in the patients’ sputum as the disease peaked. As influenza developed, characteristics of the bacilli changed. When Hall started to convalesce, they showed signs of dying and disappearing from the respiratory tract. Thus, by 6 February, with ‘the patient being much improved’, Klein noted that while he could identify ‘numerous bacteria of different species’ in Hall’s sputum, ‘it was difficult to find with certainty any but isolated Pfeiffer bacilli’. This demonstrated the pathological connection between the bacillus and the disease: before a patient passed through the height of influenza, the number of bacilli present in bronchial sputum was ‘very great’; and as the disease abated, and the patient got better, ‘the number of the bacilli also rapidly diminished’. As the bacillus disappeared, so too did influenza.^118^

Klein also linked influenza’s epidemiology to the life cycle and ecology of the bacillus. It was ‘a matter of no small practical importance’, he noted, ‘that in cases with bronchial expectoration, the *fluids of the mouth* contained an abundance of influenza bacilli’.^119^ Bacteriological examinations constituted the mouth, nose and bronchial passageways as the loci and media of infection.

Klein’s report highlighted the apparent congruence between Pfeiffer’s bacillus and influenza’s clinical and epidemiological identity. The supporting laboratory evidence was impressive. The bacillus met aetiological criteria that no other candidate had met: it was new; it was not associated with other respiratory diseases; it had a definite identity; it had unique culture requirements; its properties seemed to correspond to the clinical and epidemiological picture; and it appeared to be present in all recognisable cases.^120^ The Department sanctioned Klein’s evidence, as did the medical press. The *Lancet* profiled the bacillus’ characteristics, alleged role and importance for understanding pathogenesis and prevention.^121^ By late 1893, Pfeiffer’s bacillus was being referred to as the ‘influenza bacillus’.

## Aligning Field, Clinic and Laboratory: the New Influenza

The status of the new bacillus depended on how it conformed to the understandings of influenza in clinical and public health medicine.^122^ In clinical medicine, its reception was predictably mixed. Clinicians debated its ‘diagnostic worth’ and evaluated its ‘clinical value’.^123^ The laboratory characterisation of the bacillus was attractive because it fit the clinical characterisation of influenza. Bacteriological work supported the notion that influenza was primarily a respiratory disease. But the most vexing problem was how to connect together its clinical forms into one entity. If the bacillus was aetiologically linked to one form, as many clinicians acknowledged, did it also play a causative role in the other forms or were other agents at work?^124^

Pfeiffer offered a solution to this problem. Drawing on emerging notions of the role of toxins in bacterial infections such as tetanus and diphtheria, he attributed influenza’s forms to a *toxin* emitted by the bacillus during infection; its diffusion through the body produced constitutional symptoms.^125^ The theory made Pfeiffer’s bacillus the ‘one and only origin’ of influenza.^126^ Although the alleged ‘influenza toxin’ had not been – and never was – identified, it was promoted on the promise that it simplified influenza’s clinical picture and provided the profession with an manageable entity.

Yet, in practice, quite the opposite happened. Basing influenza’s clinical identity on the bacillus and its toxin generated classificatory frameworks which were just as complicated as their predecessors and far more esoteric.^127^ Numerous observers questioned the value of using bacteriological criteria to delineate influenza in the absence of laboratory evidence of the role of the toxin or the bacillus in different forms of the disease. Some critics doubted whether a single bacillus could be responsible for every manifestation of influenza. Others speculated that the initial respiratory infection involving Pfeiffer’s bacillus paved the way for other germs that crucially factored into the disease process. While most practitioners attributed an important role to the bacillus, rather than transform clinical frameworks it was subsumed under them.^128^

The bacillus attracted most attention as the diagnostic marker physicians so dearly wanted. Distinguishing ‘epidemic influenza’ from the various catarrhs and ‘influenza colds’ was an on-going problem.^129^ Spurred by reports from Germany that microscopic analyses of patients’ sputa could be used to redress this problem, by the late 1890s the *Lancet* and the *BMJ* advocated including bacteriological examinations in the diagnosis of influenza, as was already becoming the case for tuberculosis and diphtheria. In a review of the clinical value of influenza bacteriology in January 1899, the *Lancet* asked: ‘Can the diagnosis of influenza be based on the microscopical finding alone of the influenza bacilli’?^130^ It maintained that in certain instances identification of the microbe in the sputum was sufficient. Yet, how and when to use bacteriological tests were open to question. Since clinical identification of influenza during epidemics was relatively straightforward, most physicians reckoned that there was little need for laboratory corroboration of their diagnoses. Where bacteriological examinations were thought to be most valuable was in diagnosing uncertain cases, especially during local epidemics and inter-epidemic periods, when physicians tended to confuse influenza with other diseases. For proponents, bacteriological tests represented a way to ‘prevent the indiscriminate diagnoses …occasionally made according to particular physicians’ own ideas of influenza rather than by the recognition of a disease having definite characteristics’.^131^

But the idea of bacteriology as an arbiter of clinical diagnoses was still novel and not easily digested.^132^ Using a bacteriological examination presupposed learning and adapting to a new kind of practice. Performing the delicate work needed to render the bacillus visible demanded time, skill and the financial means to acquire equipment.^133^ Proponents argued that such obstacles were easily overcome, since the basic identification of the bacillus required only minimal experience and expense: ‘after the observer has become familiar with the appearance of the Pfeiffer bacillus under the microscope it may be readily identified in the sputum a patient suffering from influenza by direct examination without awaiting the result of cultivation’.^134^ However, fitting bacteriological examinations into clinical practice was more than just a technical problem. Since the bacillus appeared to be only pathognomonic for the respiratory influenza, physicians still had to rely on their observation skills when diagnosing other forms of the disease. Reading influenza’s symptoms continued to dominate everyday diagnostic practices. Thus, while physicians incorporated the bacillus into explanations of influenza, its laboratory identification was not readily used as a diagnostic aid.

The bacillus did, however, produce noticeable changes in the professional perceptions *and* practices of preventive medicine. Pfeiffer’s descriptions of the bacillus provided new grounds for controlling influenza. In a series of studies in 1893, he determined that while the bacillus could survive in a patient’s sputum for up to two weeks, in samples of dried sputum it survived only for two days and in cultures exposed to water or air it died within a few hours.^135^ Three important conclusions were drawn from these laboratory facts. First, the bacillus was not capable of multiplication outside the human body, either in water or in the earth. Second, the spread of influenza by dry sputum was rare. And third, moist secretions from the air passages as a rule produced infection. These properties not only put to rest any miasmatic theory, they also constituted ‘the moist secretions of the air passages’ as a specific target for preventive measures. Sir Richard Thorne-Thorne, who succeeded George Buchanan as Medical Officer to the LGB, summed up the implications of this work in late 1893:

The sputa of the sick are, especially in the acute stages of the disease, invariably charged with the microorganism which is pathognomonic of Influenza, and it may be hoped therefore that when these sputa come to be recognised as infectious and are dealt with, as is held necessary in the case of discharges from the throat, mouth and nostrils of scarlatine (sic) and diphtheria patients, the spread of Influenza from person to person may be to a corresponding extent controlled.^136^

Influenza prevention became modelled on frameworks established for tuberculosis, diphtheria and other infectious diseases. Contagion-minded epidemiologists outlined ‘human intercourse’ and the ‘assemblage of large numbers of people’ as the main pathways for spreading influenza. Bacteriologists identified the spreading agent and used laboratory knowledge of its properties to identify sneezing, coughing, spitting and talking as the principal vehicles of infection. Focus on the bacillus made it possible to monitor influenza through the minute corporeal exchanges of urban life. Public health authorities and medical officers acquired a rationale for intervening in everyday behaviours and social interactions.^137^ Influenza was now linked to enduring idea that ‘coughs and sneezes spread diseases’.

Official prevention approaches targeted the routes and places through which influenza bacillus spread.^138^ Control of the disease was based on three measures: prohibition of ‘unnecessary assemblages’ whenever an epidemic threatened; separation of the sick from the healthy; and disinfection of infected people and places.^139^ Disinfection was the key instrument. A sick person’s nasal and bronchial passageways and expectorations were washed with germ-killing solutions.^140^ Disinfection was soon proposed for groups deemed ‘susceptible’ to infection. By the turn-of-the-century, most public health authorities recommended that police officers, transit and railways workers, nurses and physicians, and school children gargle with disinfectants whenever an epidemic threatened.^141^ Patent medicines and disinfectants promising to halt the bacillus took this message to the public.^142^ Although variable in effectiveness, these practices consolidated ‘influenza bacillus’ as an object of prevention and popular knowledge, and underscored the collective experience of influenza as a democratic disease.

## Realigning Field, Clinic and Laboratory after 1900

Little laboratory work was actually done on Pfeiffer’s bacillus before 1900. But by the turn of the century, as bacteriology became increasingly institutionalised in medicine and public health, and bacteriological education created professionals trained in bacteriological methods, laboratory work on the bacillus multiplied.^143^ Yet rather than secure its relation to influenza, increased laboratory scrutiny in the 1900s and 1910s problematised its aetiological status.

Questions about its role first surfaced in 1899, when, during the largest epidemic since 1890, workers in Germany, France and the US, including Pfeiffer himself, failed to identify it in most clinical cases. The epidemic raised two issues that would loom large in the new century. The first concerned the identity of the bacillus itself. A number of researchers had identified haemophilic bacilli that were indistinguishable from Pfeiffer’s bacillus, but did not cause influenza. Found in other diseases these ‘influenza-like’ bacilli complicated bacteriological diagnosis. The second problem involved the specificity of Pfeiffer’s bacillus. While failing to find it in influenza, researchers found it in other diseases, including scarlet fever, measles and pneumonia, as well as in healthy people. Moreover, other pathogens appeared to play a role in influenza. The Cambridge University pathologist P.N. Panton summed up the difficulties: ‘While there is no doubt that this bacillus …is the causative agent of some influenzal epidemics, it must not be expected to recognise it in all cases of so-called influenza’.^144^

**Figure 11:**
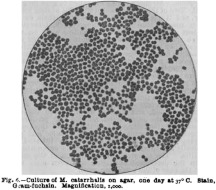
Aetiology of influenza. *Micrococcus Catarrhalis* identified by M.H. Gordon as the cause of ‘influenza’ in Hertford, January 1905. Source: R.A. Dunn & M.H. Gordon, ‘Remarks on Clinical and Bacteriological Aspects of an Epidemic Simulating Influenza which Recently Occured in East Herts district’, *BMJ*, 26 August (1905), 425.

Bacteriologists developed two contending approaches to this problem. The first, proposed by Pfeiffer, insisted that influenza be regarded as a specific disease, solely caused by his bacillus, and that clinically identical diseases caused by other organisms be called ‘pseudo-influenza’.^145^ The second suggested that influenza was not a pathological entity, but a group of diseases ‘probably…caused by different microbes’.^146^ There was already laboratory evidence to substantiate this argument. A host of other germs appeared to play an important role. One in particular, *Micrococcus catarrhalis*, captured much attention.^147^ Linked to numerous respiratory conditions, from mild catarrh to severe broncho-pneumonia, it was the most frequently isolated germ from influenza outbreaks in the early 1900s (Figure 11). Some bacteriologists suggested that it had replaced Pfeiffer’s bacillus.^148^

The combination of mounting laboratory evidence against Pfeiffer’s bacillus, and concerns over the implications of a shift in influenza’s bacteriology for medical and public health understandings, prompted moves to reframe its aetiology. Clifford Albutt, Regius Professor of Physic at Cambridge, described ‘the bacteriological position’ as ‘rather exasperating’, but concluded that, ‘the responsible microbes seem to be several’.^149^ The practical and professional consequences of this kind of explanation worried bacteriologists interested in making bacteriology indispensable to medicine. W. D’Este Emery, a clinical pathologist at King’s College Hospital, summed up the dilemma. On the one hand, drawing a distinction between ‘true’ and ‘pseudo’ influenza was of little use to practitioners: ‘the physician would hardly thank us if we told him that a patient in whom he diagnosed influenza was not suffering from that disease because Pfeiffer’s bacillus was absent, but was really ill of a disease identical in symptoms, course, event, sequelae, and treatment’.^150^ On the other hand, the idea that influenza was a multiple infection added problems to making a bacteriological diagnosis and explaining its pathogenesis. It also ran against the principle of specific aetiology. Although bacteriologists explored the notion that influenza was a ‘mixed infection’, most were unwilling to abandon this principle. Emery proposed a popular solution. Bacteriologists had to confront the possibility that they had not found the culpable pathogen. With unintended prescience, he came to the following conclusion:

The only possible solution that is entirely satisfactory is absolutely hypothetical and unsupported by any evidence. It is that influenza is a specific disease, due to a definite single cause, but that this cause is undiscovered, and perhaps unsuspected; it may be an ‘invisible’ microbe, a protozoan, or some depressing ‘influence’ acting directly on the human constitution, and of a nature as little known as were bacteria when influenza received its name.^151^

While ‘invisible’ microbes had yet to fully emerge as laboratory objects, the idea that influenza might be caused by such agents reflected the difficulty of using Pfeiffer’s bacillus to establish consensus on influenza’s identity. Nevertheless, we should not overlook its importance in establishing new ways of understanding influenza. Work on the bacillus drove the development of the bacteriology of influenza. The spread of Pfeiffer’s investigations shaped and was shaped by clinical and epidemiological knowledge, and provided the medical profession with a cause-based definition, which supplemented and, some hoped, would supplant symptom-based definitions. In clinical medicine, the microbe was a resource for explaining the pathogenesis of the disease; in public health, it was used to make visible and to target the routes of influenza transmission. Pfeiffer’s bacillus thus played an important role in aligning epidemiological, clinical and bacteriological knowledge around a new definition of influenza.

## Conclusion

Between 1890 and 1910 influenza’s medical identity had been transformed. The disease was incorporated into the disciplinary contexts of modern public health, clinical medicine and bacteriology, where new understandings were constructed of its epidemiology, symptomatology, aetiology and pathogenesis. Together, these understandings established influenza as an important modern disease. Yet, as the challenges encountered in each of these contexts demonstrate, influenza resisted classification or control by any one approach. As Clifford Albutt put it in 1907, the outpouring of epidemiological, clinical and laboratory work from 1890 underscored an essential fact: influenza was ‘of protean diseases the most protean’.^152^ While it had been drawn into the frameworks of modern medicine, its protean nature reflected and reinforced differences in epidemiological, clinical and pathological knowledge. Rather than resolve the problem of its identity, this transformation crystallised a crucial problem in modern medicine: how to align its different ways of knowing into working understandings of a disease. Establishing agreement on influenza’s medical identity would become a key challenge in the first half of the twentieth century. It was to be put to the test above all in 1918, when Britain and the rest of the world were engulfed in a devastating global pandemic.

## Footnotes

1
GreenwoodMajor, ‘The history of influenza, 1658–1911’, in Ministry of Health *Report on the Pandemic of Influenza 1918–19* (London: HMSO, 1920), 3–30: 20.

2
*Ibid.*, 23–4.

3
Ministry of Health, *Report on the Pandemic of Influenza, 1918–19* (London: HMSO, 1920), vi.

4
For the historiography and specific changes in Britain, see Michael Worboys, *Spreading Germs: Disease Theories and Medical Practice in Britain, 1865–1900* (Cambridge: Cambridge University Press, 2000), ch. 1.

5
LeichtensternOtto, ‘Influenza’, in J. Mannaberg and O. Leichtenstern (eds), *Malaria, Influenza and Dengue* (Philadelphia and London: W.B. Saunders & Co, 1905), 523.

6
*Ibid.*, 523.

7
For the revolutionary model, see Andrew Cunningham and Perry Williams (eds), *The Laboratory Revolution in Medicine* (Cambridge: Cambridge University Press, 1992); Andrew Cunningham, ‘Transforming plague: the laboratory and the identity of infectious disease’, in Andrew Cunningham and Perry Williams (eds), *idem*, 209–44. For the evolutionary model, see Christopher Lawrence, ‘Incommunicable Knowledge: Science, Technology and the Clinical Art in Britain, 1850–1914’, *Journal of Contemporary History*, 20 (1985), 503–20; J.D. Howell, *Technology and the Hospital: Transforming Patient Care in the Early Twentieth Century* (Baltimore: Johns Hopkins University Press, 1995); Michael Worboys, ‘Was There a Bacteriological Revolution in Late Nineteenth Century Medicine?’, *Studies in History and Philosophy of Biological and Biomedical Sciences*, 38 (2007), 20–42.

8
SturdySteve, ‘Looking for Trouble: Medical Science and Clinical Practice in the Historiography of Modern Medicine*Social History of Medicine*24, 3 (2011739757

9
For an overview, see Morten Hammerborg, ‘The Laboratory and the Clinic Revisited: The Introduction of Laboratory Medicine into the Bergen General Hospital, Norway’, *Social History of Medicine*, 24, 3, (2011), 758–75.

10
Olga Amsterdamska, ‘Chemistry in the clinic: the research career of Donald Dexter Van Slyke’, in S. de Chadarevian and H. Kamminga (eds), *Molecularizing Biology and Medicine: New Practices and Alliances, 1910s–1970s* (Amsterdam: Harwood Academic Publishers, 1998), 47–82. For general approaches to aligning different medical knowledge, see I. Löwy, ‘Medicine and change’, in I. Löwy and J.-P. Gaudillière (eds), *Medicine and Change: Historical and Sociological Studies of Medical Innovation* (Montrouge, France: John Libbey Eurotext, 1993), 1–19; Marc Berg, ‘Turning a Practice into a Science: Reconceptualizing Postwar Medical Practice’, *Social Studies of Science*, 25, 3 (1995), 437–76; M. Berg and Annemarie Mol (eds), *Differences in Medicine: Unraveling Practices, Techniques, and Bodies* (Durham: Duke University Press, 1999); Annemarie Mol, ‘Pathology and the clinic: an ethnographic presentation of two atheroscleroses’, in M. Lock, A. Young and A. Cambrosio (eds), *Living and Working with the New Medical Technologies. Intersections of Inquiry* (Cambridge: University of Cambridge, 2000), 82–102.

11
SchlichThomas, ‘Linking Cause and Disease in the Laboratory: Robert Koch’s Method of Superimposing Visual and Functional Representations of Bacteria*History and Philosophy of the Life Sciences*22 (20004358; Worboys, *op. cit.* (note 7).11258100

12
WorboysM., ‘From heredity to infection: tuberculosis, 1870–1890’, in GaudillièreJ.-P.LöwyI.*Heredity and Infection: The History of Disease Transmission* (LondonRoutledge200181100; Christoph Gradmann, *Laboratory Disease: Robert Koch’s Medical Bacteriology* (Baltimore: Johns Hopkins University Press, 2009).

13
LawrenceChristopher, ‘“Definite and material”: coronary thrombosis and cardiologists in the 1920s’, in RosenbergC.GoldenJ.*Framing Disease: Studies in Cultural History* (New JerseyRutgers University Press19925084

14
LöwyI.*Between Bench and Bedside: Science, Healing and Interleukin-2 in a Cancer Ward* (Cambridge, MAHarvard University Press1997

15
JacynaL.S.*Lost Words: Narratives of Language and the Brain, 1825–1926* (Princeton, NJPrinceton University Press2000

16
JacksonMark, ‘“A private line to medicine”: the clinical and laboratory contours of allergy in the early twentieth century’, in KrokerK.KeelanJ.MazumdarP.M.H.*Crafting Immunity: Working Histories of Clinical Immunology* (AldershortAshgate20085576

17
TognottiE., ‘Scientific Triumphalism and Learning from Facts: Bacteriology and the “Spanish Flu” Challenge of 1918*Social History of Medicine*16, 1 (2003971101459881910.1093/shm/16.1.97

18
PattersonD.K.*Pandemic Influenza, 1700–1900: A Study in Historical Epidemiology* (Totowa, NJRowman & Littlefield1986

19
*Ibid.*, 29–48. For contemporary accounts, see C. Creighton, *A History of Epidemics in Britain*, Vol. II (London: Cambridge University Press, 1894), 306–433 and Theophilus Thompson, *Annals of Influenza or Epidemic Catarrhal Fever in Great Britain from 1510–1837* (London: The Sydenham Society, 1852).

20
From 1861 until 1888, influenza deaths in London went above 50 once (1864); in the 1870s, they exceeded 25 once (1870); from 1880 to 1888, they averaged 7.7 per year. Registrar-General *Fifty-Fourth Annual Report of the Registrar-General, 1891* (London: HMSO, 1892), xiii–iv.

21
Ministry of Health, *op. cit.* (note 3), 23.

22
*Sixty-third Annual Report of the Registrar General, 1900*, (London: HMSO, 1902), ixvi.

23
Ministry of Health, *op. cit.* (note 3), 23–4.

24
Local Government Board, *Report on the Influenza Epidemic of 1889–1890*, Vol. XXXIV (London: HMSO, 1891); Local Government Board, *Further Report and Papers on Epidemic Influenza, 1889–92*, Vol. VLII (London: HMSO, 1893).

25
HardyA., ‘On the Cusp: Epidemiology and Bacteriology at the Local Government Board, 1890–1905*Medical History*42 (1998328346: 330–5.979657710.1017/s0025727300064012PMC1044038

26
BrandJ.L.*Doctors and the State: The British Medical Profession and Government Action in Public Health, 1870–1912* (BaltimoreJohns Hopkins Press19657375

27
*op. cit.* (note 4), 234–76.

28
‘Henry Franklin Parsons’, *BMJ*, 8 November (1913), 1263–64; ‘Henry Franklin Parsons’, *Lancet*, 18 November (1913), 1355–56. For collective investigations, see H. Marks, “‘Until the Sun of Science…the true Apollo of Medicine has risen”: Collective Investigation in Britain and America, 1880–1910’, *Medical History*, 50 (2006), 147–66.

29
ColemanWilliam*Yellow Fever in the North: The Methods of Early Epidemiology* (MadisonUniversity of Wisconsin Press1987186

30
ParsonsH.F., Local Government Board, *Report on the Influenza Epidemic of 1889–1890* (London: HMSO, 1891), 120.

31
*Ibid.*, 13.

32
ClemowF., ‘Epidemic Influenza*Public Health*24 (1890358366

33
*Lancet*, 21 December 1889, 1293.

34
DeLacyM., ‘The Conceptualization of Influenza in Eighteenth Century Britain: Specificity and Contagion*Bulletin of the History of Medicine*67, 1 (1993741188461639

35
ThompsonE.S.*“Influenza, or Epidemic Catarrhal Fever: An Historical Survey of Past”, Epidemics in Great Britain from 1510 to 1890* (LondonPercival & Co.18901723

36
See F.B. Smith, ‘The Russian Influenza in the United Kingdom’, *Social History of Medicine*, 8, 1 (1995), 62.

37
PellingM., ‘Contagion/germ theory/specificity’, in BynumW.F.PorterR.*Companion Encyclopedia of the History of Medicine* (LondonRoutledge1993309334

38
*op. cit.* (note 4), 212.

39
*op. cit.* (note 30), 78.

40
*Tenth Annual Report of the Registrar-General of Births, Deaths, and Marriages in England* (London: HMSO 1852), xxxiv; *Fourteenth Annual Report of the Registrar-General of Births, Deaths, and Marriages in England* (London: HMSO 1856), xv; A. Hirsch, *Handbook of Geographical and Historical Pathology* (London: New Sydenham Society, 1883), 18.

41
*Lancet*, 5 April (1890), 754.

42
*op. cit.* (note 30), x.

43
*Ibid.*, 52.

44
*BMJ*, 23 January (1892), 183.

45
*Lancet*, 11 July (1891), 80.

46
*BMJ*, 30 January (1892), 243–50.

47
*BMJ*, 23 January (1892), 184.

48
SisleyR.*A Study of Influenza and the Laws of England Concerning Infectious Diseases* (LondonLongman’s, Green, and Co189210.1136/bmj.1.1621.167PMC241960220753512

49
*Lancet*, 11 July (1890), 79.

50
GreenwoodM., ‘The history of influenza, 1658–1911*Report on the Pandemic of Influenza, 1918–19* (LondonHMSO1920190

51
*op. cit.* (note 1), 27.

52
*Times*, 28 December (1889), 7.

53
ParkesLouis*Hygiene and Public Health*, 3rd edn (London: H.K. Lewis, 1892), 460.

54
*op. cit.* (note 30), x.

55
*BMJ*, 23 January (1892), 184.

56
*op. cit.* (note 30), xi.

57
*op. cit.* (note 48).

58
*op. cit.* (note 30), 54.

59
PeacockT.B.*On the Influenza, or Epidemic Catarrhal Fever of 1847–1848* (LondonJ. Churchill1848); T.S. Watson, *Lectures on the Principles of Physic* (London: Longmans, Green and Co, 1871).

60
HeamanE.*St. Mary’s: the History of a London Teaching Hospital* (Montreal: McGill-Queen’s University Press, 2003), 89–118; K. Waddington, *Medical Education at St Bartholomew’s Hospital 1123–1995* (London: Boydell Press, 2003), 115–45.

61
HessV., ‘Standardizing body temperature: quantification in hospitals and daily life, 1850–1900’, in JorlandG.WeiszG.OpinelA.*Body Counts: Medical Quantification in Historical and Sociological Perspective* (MontrealMcGill-Queen’s University Press2005109126

62
GranshawL., ‘“Fame and fortune by means of bricks and mortar”: The medical profession and specialist hospitals in Britain, 1800–1948’, in GranshawL.PorterR. The Hospital in History (LondonRoutledge1989199220

63
C. Lawrence, *op. cit.* (note 7), 503–20; S. Sturdy and R. Cooter, ‘Science, Scientific Management, and the Transformation of Medicine in Britain *c*. 1870–1950’, *History of Science*, 36 (1998), 435.

64
*Lancet*, 21 December (1891), 1294.

65
PetersonM.J.*The Medical Profession in Mid-Victorian London* (BerkeleyUniversity of California Press19788889

66
*The Times* 1 January (1890), 3; *ibid.*, 7 January (1890), 5; *ibid.*, 23 January (1890), 14. For a full account of press responses, see M. Honigsbaum, ‘The Great Dread: Cultural and Psychological Impacts and Responses to the “Russian” Influenza in the United Kingdom’, *Social History of Medicine*, 23, 2 (2010), 299–319.

67
SisleyR.*Epidemic Influenza: Notes on its Origin and Method of Spread* (LondonLongmans, Green and Co.18916

68
GoodhartJ.F., ‘Influenza’, in AlbuttT.C.*A System of Medicine* (LondonMacmillan and Co.1896690

69
S. Taylor, ‘Notes on epidemic influenza’, *Lancet*, January 25 (1890), 187.

70
For diagnostic practices at Barts, see Rosemary Wall, “‘Natural”, “Normal”: Discourse and Practice at Sr. Bartholomew’s Hospital, London, and Addenbrooke’s Hospital, Cambridge, 1880–1920’, *Forum: Qualitative Social Research*, 8, 1 (2007http://nbnresolving.de/urn:nbn:de:0114-fqs0701174

71
S. West, ‘The Influenza Epidemic of 1890’, *St. Bartholomew’s Hospital Reports*, XXVI (1890), 193–258: 193.

72
*Ibid.*, 217.

73
*Ibid.*, 217.

74
See Peacock, *op. cit.* (note 59). *Quain’s Dictionary of Medicine* used Peacock’s description until 1894, when it was revised by Dawson Williams. Williams noted that an adaptation of Peacock’s original classification was ‘very widely accepted’: ‘A. *Catarrhal*’; ‘B. *Gastric*’; ‘C. *Nervous*’. See R. Quain, *A Dictionary of Medicine* (London: Longmans, Green and Co, 1894), 960.

75
ThompsonT.*Annals of Influenza or Epidemic Catarrhal Fever in Great Britain from 1510–1837* (The Sydenham Society LondonPrinted for The Sydenham1852

76
*op. cit.* (note 71), 196–7.

77
G. Smith, ‘The Influenza Epidemic’, *Transactions of the Medical Society of London*, 13, February 17 (1890), 277–306.

78
*op. cit.* (note 71), 196.

79
*The Medical Annual* (London: Simpkin, Marshall, Hamilton, Kent & Co, 1890), 529.

80
Examples can be found in Goodhart *op. cit.* (note 65); W.R. Jack, *Wheeler’s Handbook of Medicine and Therapeutics* (Edinburgh: E&S Livingstone, 1903); T.D. Savill, *A System of Medicine* (London: J&A Churchill, 1903), 645; J.W.S. Moore, ‘Influenza’, in J.W. Ballantyne (ed.), *Encyclopaedia Medica* (Edinburgh: Green & Son, 1919), 502–33.

81
See Watson, *op. cit.* (note 59) 43–6.

82
S. West, ‘An Address on Influenza’, *Lancet*, April 28 (1894), 1047–52.

83
AlthausJ.*Influenza: Its Pathology, Symptoms, Complications, and Sequels* (LondonLongmans18921320

84
RichardsonB.W., ‘Epidemic Neuroparesis*Asclepiad*9 (18921937

85
RichardsonB.W., ‘Influenza as an Organic Nervous Paresis*Asclepiad*8 (1891178179

86
HarrisW., ‘The Nervous System in Influenza’, *The Practitioner* August (1907), 85.

87
*Ibid.*, 70.

88
*op. cit.* (note 66); Smith, *op. cit.* (note 36).

89
Histories of respiratory disease have focused on tuberculosis. See F.B. Smith, *The Retreat of Tuberculosis, 1850–1950* (London: Croom Helm, 1988); S.M. Rothman, *Living in the Shadow of Death: Tuberculosis and the Social Experience of Illness in American History* (Baltimore: Johns Hopkins University Press, 1994).

90
*op. cit.* (note 71), 202.

91
*op. cit.* (note 82), 1052.

92
*Ibid.*, 1049.

93
OslerW.*The Principles and Practices of Medicine* (LondonYoung & Pentland18959293

94
*Ibid.*, 94.

95
*BMJ*, 23 January 1892, 184.

96
DonaldsonR., ‘The bacteriology of influenza – with special reference to Pfeiffer’s Bacillus’, in CrookshankF.G.*Influenza: Essays by Several Authors* (LondonWilliam Heinemann1922153

97
CrosbyA.W.*America’s Forgotten Pandemic – The Influenza of 1918* (CambridgeCambridge University Press1989269

98
See, eg., Goodhart, *op. cit.* (note 68), 697–700; A. Heywood, *Influenza: Its Causes, Cure and Prevention* (Manchester: Abel Heywood & Son, 1902); F.T. Lord, ‘Influenza’, in W. Osler (ed.), *A System of Medicine* (London: Henry Frode, 1915), 534–49.

99
*op. cit.* (note 96), 141–3.

100
*Lancet*, 9 January (1892), 99.

101
E. Klein, ‘Some Remarks on the Influenza Bacillus’, *BMJ*, 23 January (1892), 171; ‘Concerning Influenza’, *BMJ*, 23 January (1892), 184.

102
‘The Influenza Bacillus’, *BMJ*, 30 January (1892), 235.

103
‘Royal Commission on Influenza’, *BMJ*, 30 January (1892) 247; ‘The Official Influenza Inquiry’, *BMJ*, 6 February (1892), 299.

104
Parsons, Local Government BoardH.F.*Further Report and Papers on Epidemic Influenza, 1889–92* (LondonHMSO1893x

105
See Worboys, *op. cit.* (note 4), 211–2.

106
Mendelsohn and Gradmann respectively have developed this argument. J.A. Mendelsohn, ‘Cultures of Bacteriology: Formation and Transformation of a Science in France and Germany, 1870–1914’, (unpublished PhD Dissertation, Princeton, 1996); C. Gradmann, ‘A Harmony of Illusions: Clinical and Experimental Testing of Robert Koch’s Tuberculin, 1890–1900’, *Studies in History and Philosophy of Biological and Biomedical Sciences*, 35, 3 (2004), 465–81.10.1016/j.shpsc.2011.10.00122326067

107
F.W. Andrewes, ‘The Work and Needs of the Pathological Department’, *St Bartholomew’s Hospital Journal*, 11 (1904), 105–9; *idem*, ‘The Beginnings of Bacteriology at Barts’, *St Bartholomew’s Hospital Journal*, 35 (1928), 100–4: 116–7.

108
KleinE., ‘Report on influenza in its clinical and pathological aspects*Further Report and Papers on Epidemic Influenza, 1889–92* (LondonHMSO189385

109
PfeifferR.BeckM., ‘Weitere Mittheilungen über den Influenza-Erreger*Deutsche Medicinische Wochnenschrift*18 (1892465

110
R. Pfeiffer, ‘Preliminary Communication on the Exciting Causes of Influenza’, *BMJ*, 16 January (1892), 128.

111
S. Kitasato, ‘On the Influenza Bacillus and the Mode of Cultivating it’, *BMJ*, 16 January (1892), 128.

112
‘Epitome of Current Literature’, *BMJ*, 25 June (1892), 104; Klein, *op. cit.* (note 108), 93.

113
Pfeiffer and Beck, *op. cit.* (note 109).

114
*op. cit.* (note 108), 119.

115
*Ibid.*, 266–70.

116
*Ibid.*, 150.

117
*Ibid.*, 120–1.

118
*Ibid.*, 121.

119
*Ibid.*, 121.

120
These criteria were summed up in Klein’s 1893 report.

121
‘The Etiology of Influenza’, *Lancet*, 8 April (1893), 810.

122
*op. cit.* (note 4), 211–2, has argued that a key factor in British doctors’ reception of Koch’s tubercle bacillus was ‘the congruence between the reported properties of the *bacillus* and their clinical experience of the disease syndrome’.

123
‘The Bacillus of Influenza’, *Lancet*, 20 May (1899), 1378–9.

124
‘The Influenza Epidemic – Report of the Medical Department of the Local Government Board’, *BMJ*, 26 August (1893), 488.

125
PfeifferR., ‘Die Aetiologie der Influenza*Zeitschrift für Hygiene*12 (1893357386

126
*Lancet*, 12 February (1898), 449.

127
*op. cit.*, (note 5), 591–2.

128
T.C. Allbutt, ‘Influenza’, *The Practitioner*, LXXVII, (1907), 1–10; W. Gray, *Influenza, with Special Reference to Some Peculiar Symptoms* (London: H.K. Lewis, 1897).

129
*Lancet*, 18 March (1899), 778.

130
*Lancet*, 14 January (1899), 103.

131
*Lancet*, 14 January (1899), 103.

132
JacynaL.S., ‘The Laboratory and the Clinic: The Impact of Pathology on Surgical Diagnosis in the Glasgow Western Infirmary, 1875–1910*Bulletin of the History of Medicine*62 (19883844063067788

133
DigbyA.*The Evolution of British General Practice, 1850–1948* (OxfordOxford University Press199953ff

134
*Lancet*, 18 March (1899), 778.

135
*op. cit.* (note 122), 357–86.

136
*op. cit.* (note 101), ix.

137
For this process in relation to diphtheria, see C. Hooker and A. Bashford, ‘Diphtheria and Australian Public Health: Bacteriology and its Complex Applications, *c*. 1890–1930’, *Medical History*, 46 (2002), 41–64.

138
Local Government Board, *Provisional Memorandum upon Precautions Advisable at Times when Epidemic Influenza Threatens or is Prevalent* (London: HMSO, 1892), xi.

139
*Ibid.*, xi.

140
*Ibid.*, xi.

141
Local Government Board, *Lancet*, 20 January (1906), 180–2.

142
LoebL., ‘Beating the Flu: Orthodox and Commercial Responses to Influenza in Britain, 1889–1919*Social History of Medicine*18, 2 (2005203224

143
The bacillus first appeared in British bacteriology textbooks in 1896. See E. Klein, *Microorganisms and Disease: An Introduction into the Study of Specific Micro-organisms* (London: Macmillan and Co, 1896) and E.M. Crookshank, *A Textbook of Bacteriology* (London: H.K. Lewis, 1896).

144
PantonP.N.*Clinical Pathology* (LondonJ&A Churchill1913133

145
W.D.E. Emery, ‘The Micro-organisms of Influenza’, *The Practitioner*, LXXVII, (1907), 109–17.

146
‘Discussion on Influenza’, *BMJ*, 13 May (1905), 1044–45.

147
*Micrococcus catarrhalis*, see A. Besson, *Practical Bacteriology, Microbiology and Serum Therapy (Medical and Veterinary): A Textbook for Laboratory Use* (London: Longmans, Green and Co., 1913), 504.

148
‘Discussion on Influenza’, *BMJ*, 13 May (1905), 1044–5.

149
*op. cit.* (note 128), 2.

150
*op. cit.* (note 145), 114–5.

151
*Ibid.*, 116–7.

152
*op. cit.* (note 125), 1.

